# Automated analysis for multiplet identification from ultra-high resolution 2D- ^1^H, ^13^C-HSQC NMR spectra

**DOI:** 10.12688/wellcomeopenres.18248.1

**Published:** 2022-10-14

**Authors:** Laura Ferrante, Kashif Rajpoot, Mark Jeeves, Christian Ludwig

**Affiliations:** 1 School of Computer Sciences, University of Birmingham, Birmingham, B15 2TT, UK; 2 University of Birmingham Dubai, Dubai International Academic City, United Arab Emirates; 3 Institute of Cancer and Genomic Sciences, University of Birmingham, Birmingham, B15 2TT, UK; 4 Institute of Metabolism and Systems Research, University of Birmingham, Birmingham, B15 2TT, UK

**Keywords:** Metabolism, metabolic tracing, NMR spectroscopy, independent component analysis, machine learning, automated

## Abstract

**Background: **Metabolism is essential for cell survival and proliferation. A deep understanding of the metabolic network and its regulatory processes is often vital to understand and overcome disease. Stable isotope tracing of metabolism using nuclear magnetic resonance (NMR) and mass spectrometry (MS) is a powerful tool to derive mechanistic information of metabolic network activity. However, to retrieve meaningful information, automated tools are urgently needed to analyse these complex spectra and eliminate the bias introduced by manual analysis. Here,

we present a data-driven algorithm to automatically annotate and analyse NMR signal multiplets in 2D- ^1^H, ^13^C-HSQC NMR spectra arising from ^13^C - ^13^C scalar couplings. The algorithm minimises the need for user input to guide the analysis of 2D- ^1^H, ^13^C-HSQC NMR spectra by performing automated peak picking and multiplet analysis. This enables non-NMR specialists to use this technology. The algorithm has been integrated into the existing MetaboLab software package.

**Methods: **To evaluate the algorithm performance two criteria are tested: is the peak correctly annotated and secondly how confident is the algorithm with its analysis. For the latter a coefficient of determination is introduced. Three datasets were used for testing. The first was to test reproducibility with three biological replicates, the second tested the robustness of the algorithm for different amounts of scaling of the apparent J-coupling constants and the third focused on different sampling amounts.

**Results: **The algorithm annotated overall >90% of NMR signals correctly with average coefficient of determination ρ of 94.06 ± 5.08%, 95.47 ± 7.20% and 80.47 ± 20.98% respectively.

**Conclusions:** Our results indicate that the proposed algorithm accurately identifies and analyses NMR signal multiplets in ultra-high resolution 2D- ^1^H, ^13^C-HSQC NMR spectra. It is robust to signal splitting enhancement and up to 25% of non-uniform sampling.

## Introduction

An efficient metabolism is the basis for the survival of any living organism ^1, 2^. Inside each cell, the network of metabolic reactions is organised into discrete but intrinsically linked metabolic pathways. Inside the cell, these pathways then function within different cellular compartments. Just as healthy metabolism requires the efficient functioning of the metabolic network, perturbations within one compartment can result in disease by spreading through and altering the entire metabolic network ^3, 4^. This is a direct consequence of inherent redundancy of the metabolic network, which means that many metabolites can be anabolised or catabolised by several different metabolic pathways.

Metabolomics studies allow the measurement of the concentrations of metabolites present at a given time, but provide no direct information as to the metabolic source of these metabolites. Feeding metabolic precursors enriched with low-abundance stable isotopes such as ^13^C, for example [1,2- ^13^C] glucose or [U- ^13^C] glutamine, enables the tracing of specific metabolic pathways usage. The contribution of the different metabolic pathways leading to the production of this metabolite can be untangled through the analysis of the ^13^C distribution within the metabolite ^5– 7^.

While technologies such as gas chromatography–mass spectrometry (GC-MS) are highly sensitive but low-resolution, nuclear magnetic resonance (NMR) spectroscopy is a high-resolution technique, albeit with relatively low sensitivity. In our previous work, we developed and published a method for the combined analysis of NMR spectroscopic and MS data that harnesses the strengths of both technologies to produce highly-resolved metabolism information in the form of metabolite isotopomers ^8^. The 2D- ^1^H, ^13^C-HSQC NMR spectrum contains information about the relative incorporation of ^13^C labelled nuclei into metabolites. This information is found in the multiplets of each resonance, with the pattern of the multiplet being due to the presence or absence of ^13^C nuclei in positions adjacent to the detected nuclei. The extent of peak splitting due to isotope incorporation (known as J-coupling) is dependent on the nuclei involved and the local chemical structure. Each multiplet is therefore virtually unique for a specific resonance of each metabolite. As many different combinations of labelling patterns may be present in each sample, the overall peaks pattern can be complex but can always be derived as a linear superposition of the individual multiplet components.

**Figure 1.**
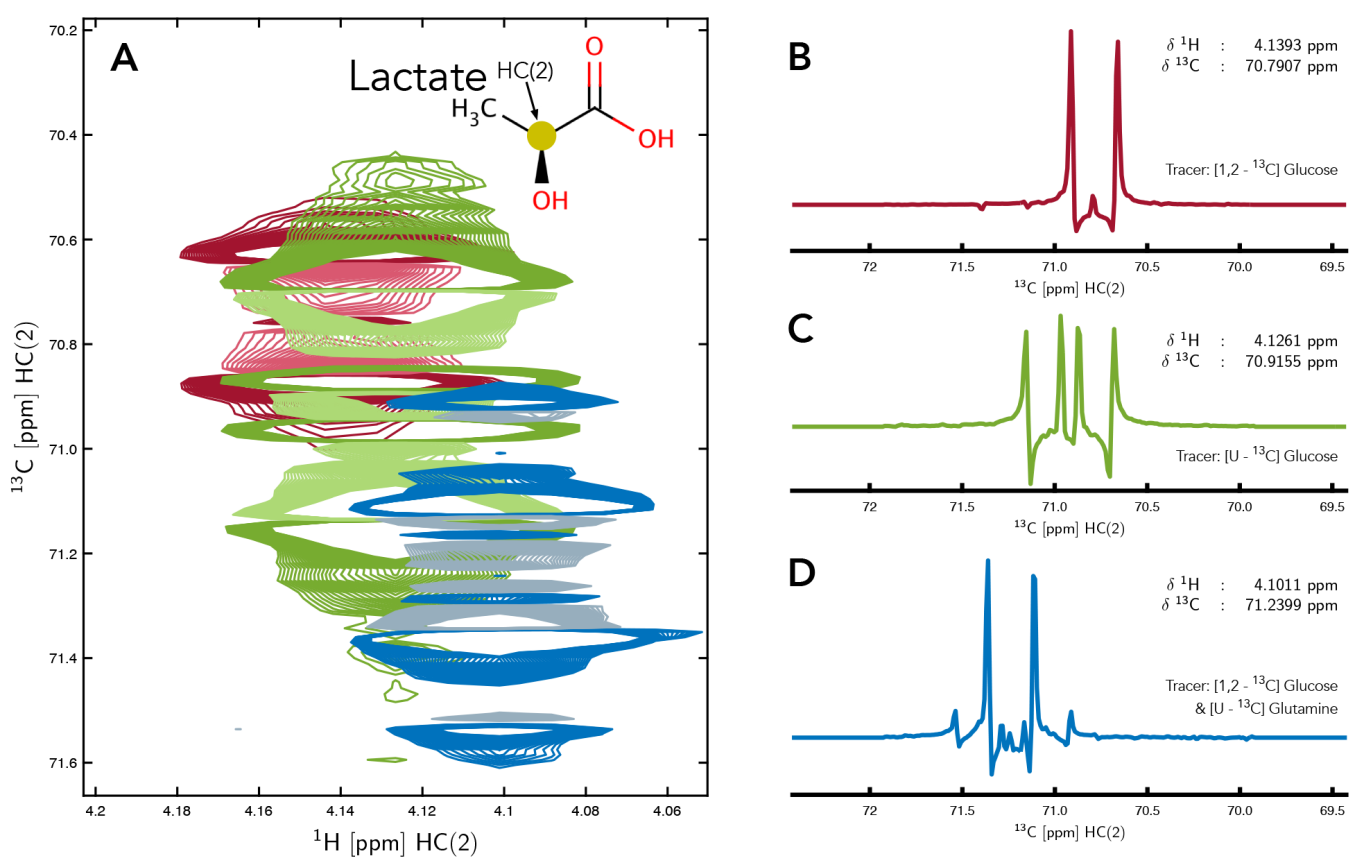
Resonance of HC(2) of lactate in 2D- ^1^H, ^13^C-HSQC NMR spectra from three different samples demonstrating the variation that can occur between samples even when observing the ^13^C multiplet of the same carbon nucleus (panel **A**). For the sample producing the red spectrum [1,2- ^13^C] glucose was used, for the green spectrum [U- ^13^C] glucose and for the sample of the blue spectrum, a mixture of [1,2- ^13^C] glucose and [U- ^13^C] glutamine were used as metabolic tracer. 1D ^13^C slices, extracted from the middle of each 2D spectrum are also shown to clearly demonstrate the differences in the appearance of the multiplets resulting from the different choice of tracers (panels **B**, **C** & **D**).

Signal annotation and multiplet analysis are the biggest challenges in the analysis of such 2D- ^1^H, ^13^C-HSQC NMR spectra, as precise resonance positions of metabolite resonances can change as a result of minor pH or sample composition changes. This can provide significant challenges in crowded areas of the NMR spectrum, especially for multiplet signals with similar, albeit distinct ^13^C/ ^13^C-Jconstants. In addition, the overall shape of a specific multiplet differs quite drastically depending on the choice of tracer (e.g. [U- ^13^C] vs [1,2- ^13^C] glucose), as well as the specific activity of metabolic pathways. An example of these challenges is depicted in Figure 1, where the signal for HC(2) of lactate is shown for three different samples of different tracers. In addition, the chemical shifts of the three lactate signals are different for the three samples because of differences in pH. It becomes quite clear why the analysis of such 2D- ^1^H, ^13^C-HSQC NMR spectra requires an in-depth knowledge and expertise in 2D- ^1^H, ^13^C-HSQC NMR spectroscopy of complex mixtures in order to perform an efficient and an unbiased analysis of the data. Automated data analysis of 2D- ^1^H, ^13^C-HSQC NMR tracing data provides an avenue free of operator bias and allows researchers with limited NMR and data analysis expertise to use this powerful analytical technique with ease.

The necessity for automated algorithms has been recognised in other NMR spectroscopy areas such as automatic assignment and data analysis for metabolomics applications ^9, 10^ and machine learning applications for HSQC data analysis for drug discovery ^11, 12^. However, no such approaches have been implemented so far for the automated analysis of ^13^C -multiplets present in 2D- ^1^H, ^13^C-HSQC NMR spectra used for metabolic tracing NMR experiments.

Here, we present a machine-learning based algorithm, which automatically annotates and analyses multiplets in 2D- ^1^H, ^13^C-HSQC NMR. After signal annotation and multiplet decomposition, the algorithm assesses quality of data analysis, which will inform the user about how much trust they can put into the result. The software developed here is fully integrated in the MetaboLab software package ^13^ and can be run as a fully automated procedure to be used by researchers with biological expertise without the necessity of expert NMR or data analysis knowledge.

## Algorithm development

### Outline

Mathematically, the identification of a metabolite signal presents itself as a signal unmixing problem. The aim is the decomposition of the NMR spectrum into statistically independent components by using a data-driven approach and without prior knowledge on how the multiplet components mix in the 2D spectrum. A 2D- ^1^H, ^13^C-HSQC NMR spectrum is a linear superposition of all resonances from each ^13^CH pair in each metabolite within the sample. The NMR multiplets of each metabolite are independent of the resonances of all other metabolites. Thus, it is theoretically possible to disentangle major spectrum components in a data-driven manner. The problem can be formulated as an inverse modelling problem, where multivariate observations (e.g. the 2D- ^1^H, ^13^C-HSQC NMR spectrum) relate to lower-dimensional vectors of statistically independent variables (e.g. the contribution of each metabolite to the 2D- ^1^H, ^13^C-HSQC NMR spectrum), using a linear model. Once a metabolite multiplet has been identified and localised within the spectrum, the contribution of each multiplet component to the entire signal must be quantified. The algorithm presented here uses linear least square regression (LS) with parameters constrained to non-negative values only ^14^ to estimate the relative contribution of each multiplet component to the experimentally found resonance.

**Figure 2.**
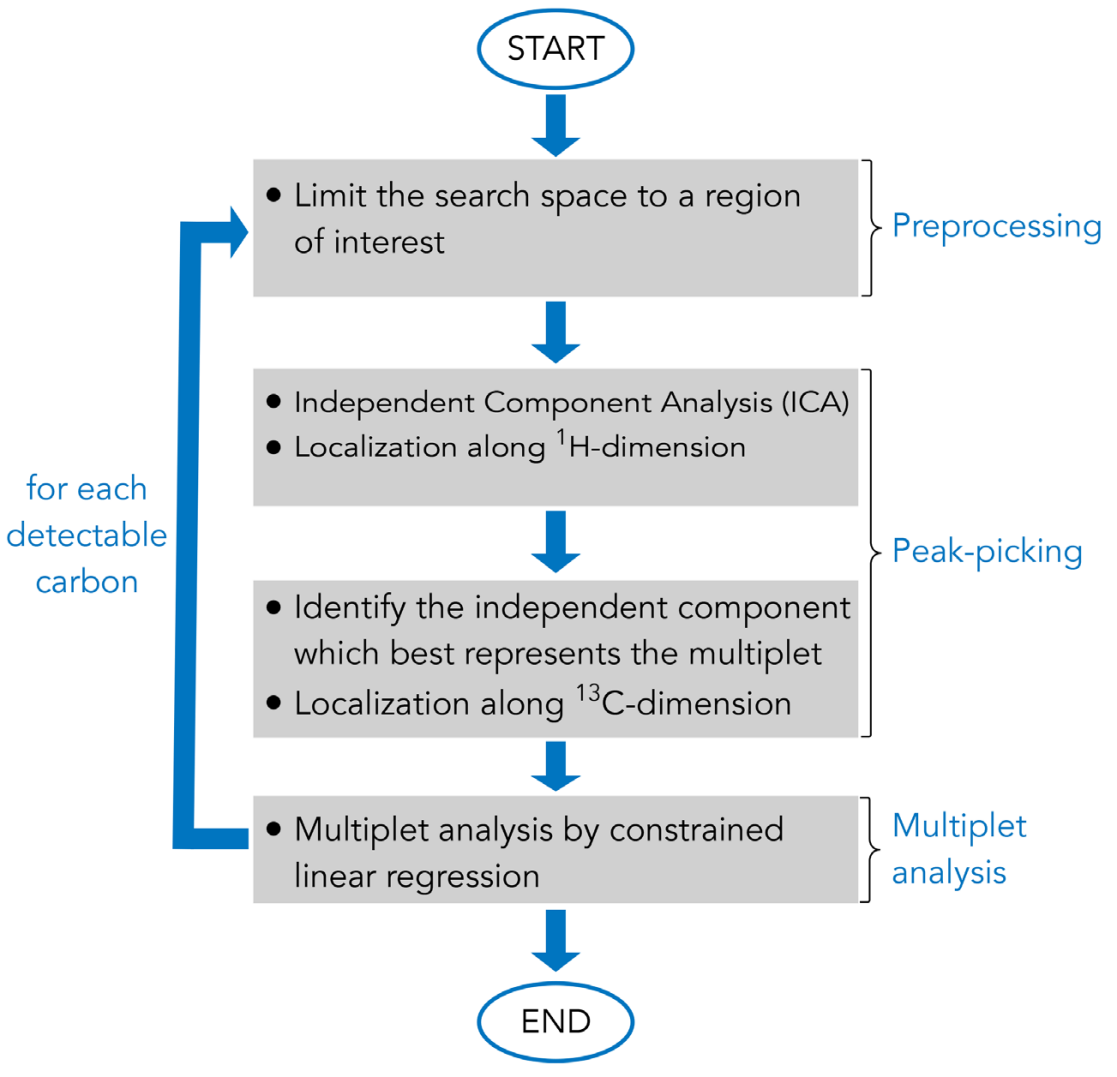
Schematic representation of the main steps of the algorithm for automated 2D- ^1^H, ^13^C-HSQC NMR spectrum analysis. The computational routine uses a list of metabolite names as input and outputs a metabolite compositions as well as the software reliability for each resonance analysis. Optionally, the user can choose to produce a graphical report of the analysis in form of a pdf file.

As a result, in order to implement an algorithm capable of automated signal annotation and multiplet decomposition, the computational problem for each resonance of each metabolite was subdivided into several steps ( Figure 2):

1. Restrict the searching area of the resonance of interest in the NMR spectrum based on a 2D- ^1^H, ^13^C-HSQC NMR spectral database built into the MetaboLab software
2. Run the Independent Component Analysis (ICA) to obtain the latent factors (e.g. ^13^C spectra of any resonance present in the restricted area) and localise each latent component along the proton dimension
3. Identify the independent component representative of the multiplet of interest and precisely determine the ^1^H and ^13^C chemical shifts
4. Perform the multiplet analysis by constrained linear regression and compute the coefficient of determination *ρ* (%) to guide the user in the understanding of the software outputs.

In the following sections, the pipeline of the algorithm developed in this work is presented, each section explains a principal element of the algorithm ( Figure 2). To provide an overview, a pseudo-code representation of the implemented algorithm can be found in the extended data repository (githubRef, Table S1 and Algorithm 1).

### Metabolite localisation in the HSQC NMR spectra

The assignment of NMR resonances to specific metabolite nuclei is based on a peak list obtained from a spectral library included in the MetaboLab software which was derived from publicly available databases ^15– 18^.

For a given metabolite, the algorithm loops through each resonance and derives expected ^1^H and ^13^C chemical shifts from the built-in database. The next logical step would be to identify the precise location of the spectral resonance. However, because this algorithm works without any *a priori* knowledge about metabolic pathways or which metabolic tracer was used, we do not have any knowledge about neither the exact chemical shift values of the resonance nor the multiplet composition. In order to identify the correct multiplet in the 2D- ^1^H, ^13^C-HSQC NMR spectrum, we can use information about ^13^C/ ^13^C-Jcouplings, stored in the spectral database included with the software. While differences in pH and sample matrix composition may lead to a change in the precise resonance location in the 2D- ^1^H, ^13^C-HSQC NMR spectrum, the ^1^H and ^13^C chemical shift values will still be similar to the values given by the database. To improve the software’s computational efficiency, the search space (i.e. the portion of 2D- ^1^H, ^13^C-HSQC NMR spectrum to be examined) for each multiplet signal is restricted from the entire spectrum to an area of *±*maxWidth1H ppm ( *±*0.15 ppm) and *±*maxWidth13C ppm ( *±*3.0 ppm) around the ^1^H and ^13^C library shift of the metabolite respectively. The maxWidth parameters refer to user changeable parameters with the standard values indicated if the user does not provide these values. This range allows the algorithm to accommodate changes in chemical shift due to the aforementioned factors.

### Multiplet identification: Independent Component Analysis (ICA)

As mentioned earlier, the computational problem to identify a particular metabolite multiplet in the restricted spectral area can be understood to be a signal unmixing problem. One particularly efficient way to solve this problem is **I**ndependent **C**omponent **A**analysis (ICA). Mathematically, we perceive the NMR spectrum columns (i.e. ^13^C 1D sub-spectra) of the chosen spectral area to be *N* observations ( **y**_1_( *ω*), **y**_2_( *ω*),.., **y** *_i_* ( *ω*),.., **y** *_N_* ( *ω*)), where each observation **y** *_i_* ( *ω*) ∈ ℝ *^T^* contains contributions of a smaller number of *K* NMR signals and noise. The *K* number of signals are also known as latent variables ( **s**_1_( *ω*), **s**_2_( *ω*),.., **s** *_i_* ( *ω*),.., **s** *K* ( *ω*)). While the noise of each spectral column is assumed to be independent, we assume each observation **y** *_i_* ( *ω*) to be a mixture of *K* latent variables weighted by unknown coefficients. We can then define a mathematical model as follows, using a vector-matrix notation and dropping the frequency dependence *ω* without loss of generality:

Y=WS(1)

where **Y** ∈ ℝ *^N ×T^* is the observation matrix, **W** ∈ ℝ *^N ×K^* is the unknown linear mapping matrix from the latent space **S** ∈ ℝ *^K ×T^* to the observation space.

In the case of a 2D- ^1^H, ^13^C-HSQC NMR spectrum of a metabolite mixture, all the multiplets of the metabolites within the restricted spectrum area are latent variables ( **s**_1_, ..., **s** *_T_* ) and the restricted area of the 2D- ^1^H, ^13^C-HSQC NMR spectrum is the observation. While a 1D-ICA generally requires multiple 1-dimensional observations, here one 2-dimensional observation is available (i.e. the search area of the 2D- ^1^H, ^13^C-HSQC NMR spectrum).

**Figure 3.**
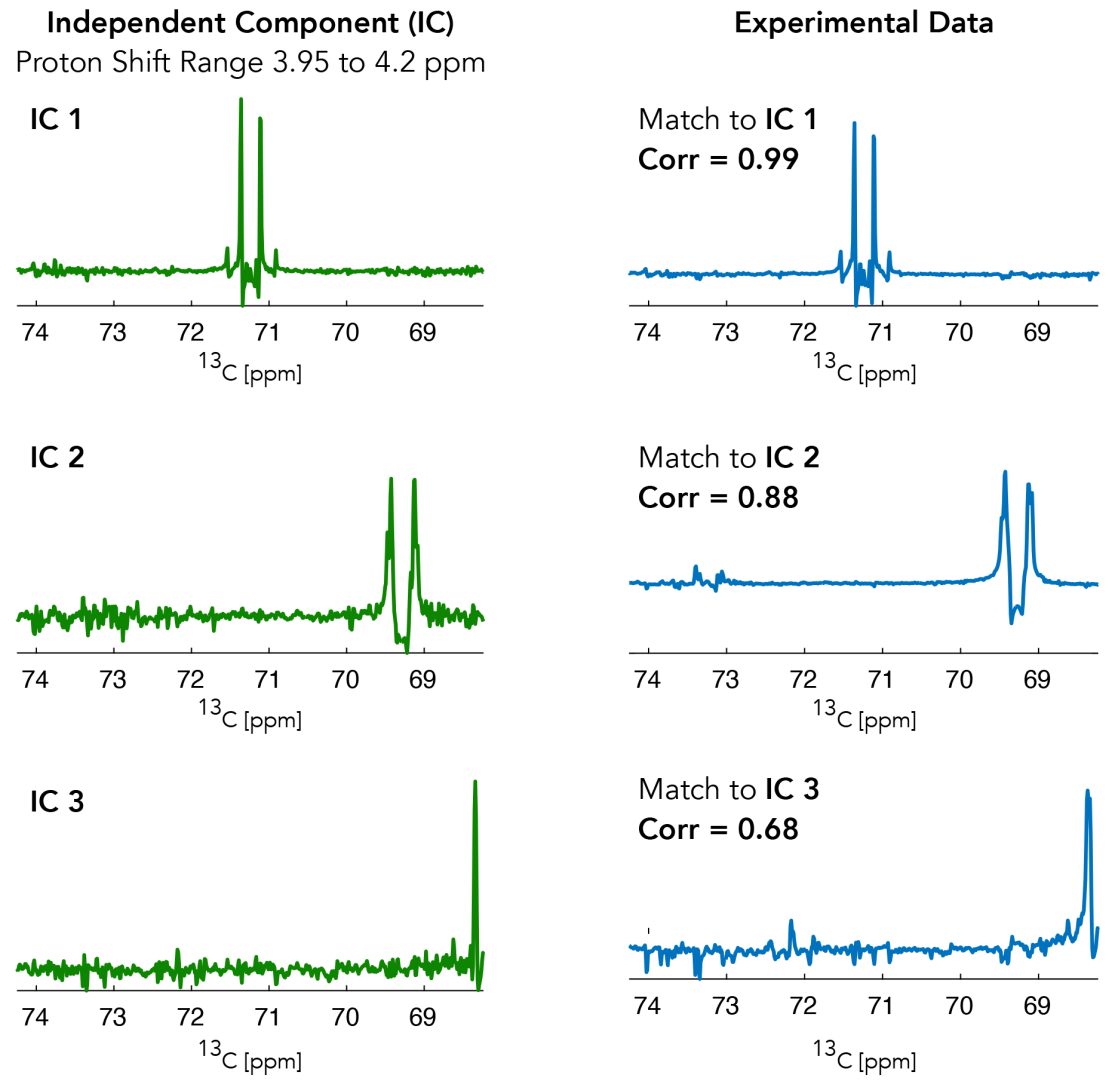
Independent components from the region around the expected shifts of HC(2) of lactate. These independent components are then matched back to the experimental data with the highest correlation component being selected.

The shortcoming of having only a single 2D observation is addressed by utilising the proton dimension as the observation dimension. This means that all the ^13^C NMR spectra at different proton chemical shifts of the 2D-spectrum are considered as observations, therefore the proton dimension is the observation dimension, and the problem can be solved using 1D-ICA. The K latent factors are the underlying spectral components (e.g. the mutiplets of the metabolites). The K components are assumed to be statistically independent as required for the ICA algorithm ^19^. This assumption is also physically/chemically plausible, as there is no interaction between metabolites at different ^1^H-shifts ^20^. Moreover, the linearity of the model can be justified, as the metabolites mix in an additive way in the spectrum ^20^. These assumptions justify the choice of using ICA among other unmixing algorithms such as non-negative matrix decomposition (NMF), since ICA finds a decomposition of the observed data to retrieve latent components which are as independent as possible. For the practical implementation of this algorithm, we chose to use an implementation of the FastICA ^21^ algorithm. Details on the working principle of 1D-ICA and its adaptation to solve the proposed problem are described in the Supporting Information (Section: *Independent Component Analysis for mutiplets identification*). Figure 3 provides an example of some of the ICA components (independent components are shown in green and experimental ^13^C NMR sub-spectra are plotted in blue) provided by the ICA algorithm during the analysis for HC(2) of Lactate. The ^1^H chemical shift range for the shown independent components was 3.95 to 4.2 ppm.

### Localisation along the proton dimension by cross-correlation

While ICA is very successful in identifying statistically independent resonances in the ^13^C dimension, information about the ^1^H localisation is lost. In order to assign a specific ^1^H chemical shift to each latent component a cross-correlation between each component and all columns from the search area of the experimental NMR spectrum is required. Because each of the NMR columns is associated with a specific ^1^H ppm value, the column which receives the largest cross-correlation value determines the assigned ^1^H chemical shift of that component. To avoid selection of latent components representing spectral noise only, the algorithm uses a minimum threshold for the correlation value ( *minCorr*) below which the latent component will be discarded from further evaluation. The standard value of this minimum correlation is 0.8, which can be adjusted by the user if needed.

### Latent component identification

**Figure 4.**
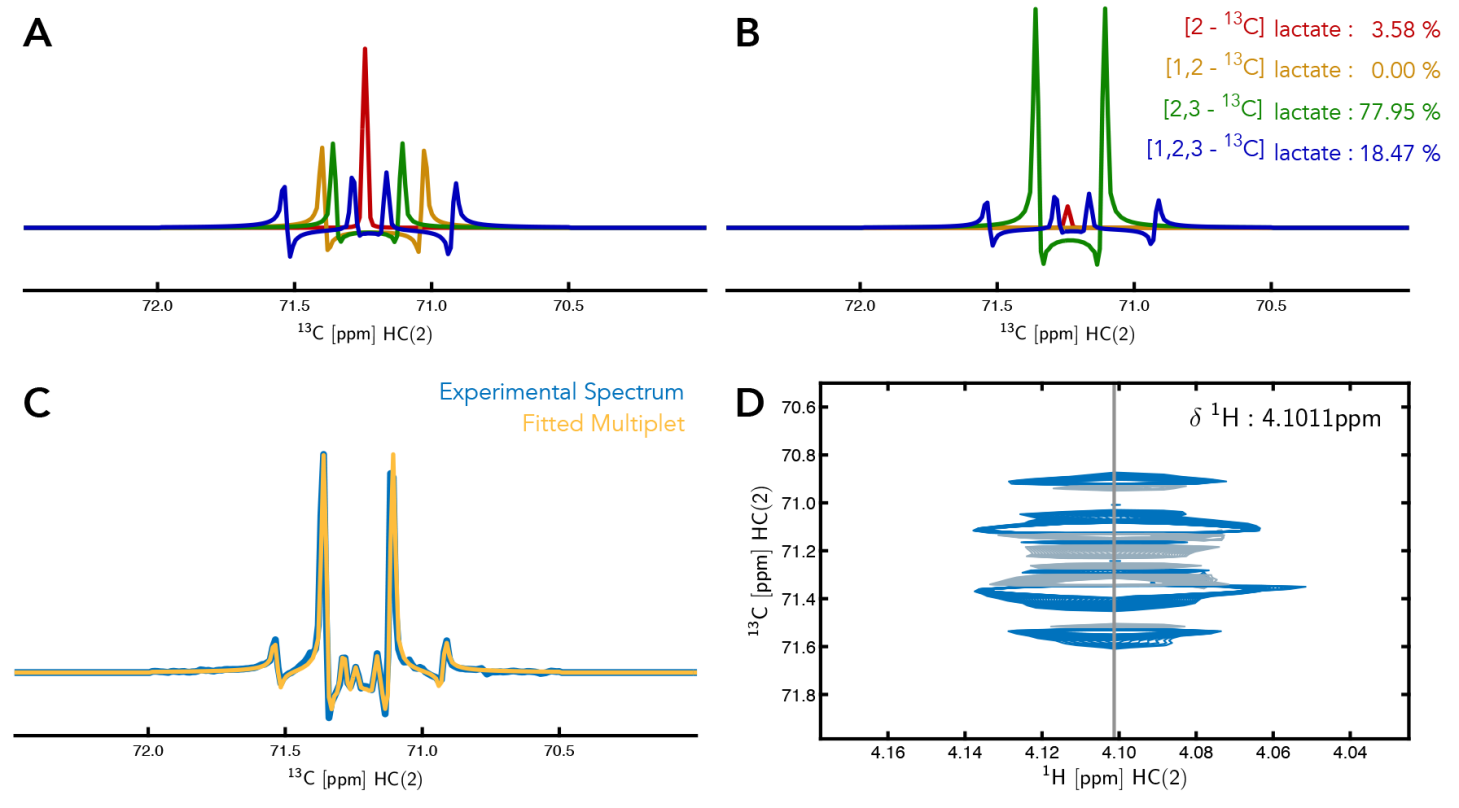
Multiplet analysis of Lactate C(2). ( **A**) The individual components that make up the multiplet of C(2) of lactate. ( **B**) Simulated individual components of C(2) of lactate with the percentage contribution of each component to the multiplet signal of ^13^C-labelled Lactate C(2). ( **C**) An overlay of the experimental data and the fitted simulated data based on the calculated percentage contributions of each labelled form of lactate. ( **D**) Region of the 2D- ^1^H, ^13^C-HSQC spectrum showing the multiplet peak for lactate C(2).

The latent components localised within the spectrum might have captured multiplet patterns of metabolites within the searching area, which will contain the resonance of the metabolites of interest if this actually exists in the current spectrum. However, these components will also contain resonances belonging to CH pairs of other metabolites. To determine if the resonance of interest exists in one of the estimated latent components, the following steps are necessary:

1. The multiplet of interest is simulated using chemical shift values and ^13^C/ ^13^C-J-coupling constants stored in MetaboLab’s database are used assuming equal intensities for each of the multiplet components as shown in Figure 4 panel A. The pygamma NMR simulation library (version 4.3.4) is used for multiplet simulations ^22^.
2. This multiplet is then aligned with each latent component through cross-correlation (of the simulated multiplet with the latent components) and the ^13^C chemical shift value of the simulated multiplet for the latent component with the highest cross-correlation value is chosen.

- This alignment is not trivial since the magnitude of the multiplet components is unknown at this point of the pipeline and alignment methods based on cross-correlation are sensitive to difference in signals’ magnitude. Moreover, presence of "outlier" peaks (peaks belonging to another metabolite or noise) pose additional challenges.
- To solve this problem, all possible combinations of simulated multiplets are considered. For example, if we consider a total of *p* = 4 multiplet components (i.e. we neglect long-range ^13^C/ ^13^C-J-couplings) m and we choose a subset of q component at a time, this computation will result in $\frac{p!}{(p-q)!q!}$ combinations. Each of these combinations is aligned with the latent component and a regression analysis is performed to obtain the coefficient of determination *ρ* scores, which quantifies the goodness of the fitting. The combination of multiplet components which gives the highest *ρ* value is chosen and the *ρ* score saved. Theoretically, this should result in the highest *ρ* score for the correct multiplet simulation at the right ^13^C chemical shift.
- However, the *ρ* value is sensitive to underlying noise and NMR resonances close to the resonance of interest. Therefore, in each latent components only NMR signals with maxima greater than 50% of the global maximum within the search area are retained and noise related issues can be avoided. In addition, the score *ρ* is weighted by the squared deviation ∆ *ppm* of the determined ^13^C chemical shift ( *x _H_*, *x _C_*) from the library ^13^C chemical shift ( *x _Hlib_*, *x _Clib_*) computed as Euclidean distance ( Equations 2 – Equations 4) ^23^.

$$\Gamma_{adjusted}=20\frac{\gamma_{H}}{\gamma_{C}}\;(2)$$

$$\Delta ppm=\sqrt{{\left(x_{H}-x_{Hlib}\right)}^{2}+{\left(\frac{x_{C}-x_{Clib}}{\Gamma_{adjusted}}\right)}^{2}}\;(3)$$

$$\rho_{adjusted}=\frac{\rho }{\Delta ppm^{2}}\;(4)$$

where *γ _H_* and *γ _C_* are the ^1^H and ^13^C gyromagnetic ratios.

### Multiplet analysis: contribution of multiplet components to overall signal

As the last step, the relative intensity of each multiplet component is then determined by a constrained linear least square regression. Prior to this step we optimize the alignment along the proton dimension using the hillclimbing heuristic optimization algorithm ^24^ to ensure that the ^1^H chemical shift obtained in the previous localisation step corresponds to the maximum of the experimental NMR resonance in the ^1^H dimension. The regression is defined as follows 5:


f(X;B)=F=XB(5)

where *F* is the simulated multiplet with adjusted intensities of all multiplet components, ***X*** is a matrix containing the different multiplet sub-spectra as input and ***B*** ≥ 0 is a vector containing the relative contribution values, which is the vector of unknowns that needs to be determined. After the constrained linear regression, vector ***B*** is normalised, so that all relative contributions add up to be 1.

## Experimental methods

### Sample preparation

For the test NMR samples, cells were cultured in the presence of isotopically labelled tracer, chosen dependent on the pathway of interest. For instance, cells cultured in the presence of [1,2- ^13^C 2]-D-glucose can provide information regarding flux through pyruvate dehydrogenase (PDH) and into the tricarboxylic acid (TCA) cycle via the isotomomer composition of C4 of glutamate. Metabolites were extracted using a biphasic system to remove proteins and lipophilic molecules whilst retaining the polar molecules ^25^.

### NMR methodology

2D- ^1^H, ^13^C-HSQC NMR spectra were collected using the standard procedures described previously ^8, 26, 27^. Spectra were acquired on either a Bruker NEO 800-MHz or a Bruker Avance 600-MHz spectrometer. Spectrometers were equipped with 1.7mm z-PFG TCI CryoProbes. All 2D-HSQC NMR spectra were acquired using echo/anti-echo gradient selection with a presaturation pulse during the 1.5s interscan delay to suppress the water resonance. Typically, spectra were acquired using 2 scans, 512 complex data points and a spectral width of 15.6ppm in the ^1^H dimension. The ^13^C dimension was acquired using 25% of 8,192 complex data points and a spectral width of 189.8 ppm resulting in an experiment time of approximately 4 hours. To test the efficacy of the algorithm with regards to the scaling of the splitting due to J-couplings experiments using scaling of 0, 2, 4 or 8 were collected. The non-uniformly collected spectra were reconstructed using the IRLS algorithm using 20 iterations with MDDNMR (version 2.5) ^28, 29^ and then processed using NMRPipe (Version 9.2) ^30^. A polynomial baseline correction was applied following manual phase correction.

### Implementation

While the biggest part of the algorithm is implemented in MATLAB (source code is available here: https://zenodo.org/record/7120367#.YzR6FC0w30o), the multiplet simulation is performed within python (version 3.9) using the pygamma NMR simulation library (version 4.3.4, https://pypi.org/project/pygamma/) ^22^. In addition the fastICA package is needed, which is available at https://research.ics.aalto.fi/ica/fastica/. The MATLAB version used was 9.10.0.1739362 (R2021a). The MetaboLab version was 2022.0726.1733 (available at https://www.ludwiglab.org/software-development). To perform the automated HSQC multiplet analysis, the The MetaboLab scripting interface is used. We provide examples of all the scripts used to analyse the three different data sets presented here as part of the extended data pdf document, available here: 10.5281/zenodo.7120367 ^31^. The datasets used as discussed in the Underlying data section ^32, 33^. 

### Operation

The algorithm is tested on a laptop with an Intel i7-6700HQ 2.60GHz CPU and a GTX 960M GPU. The analysis of a single spectrum is performed in less than 5 minutes.

## Results and discussion

### Program output

While all resulting information is stored inside the MetaboLab data structure, the user has the option to create a clear text report in the form of a pdf file ( report: on). This report (see Supporting Information) contains sample information originally stored inside TopSpin’s title file, a list of the metabolites analysed and the user defined variables for the multiplet assignment and analysis ( maxWidth1H, maxWidth13C, minCorr, maxRange, nReps, R2, R2 (scaled), dataSets, experiments). The following information is output for each observable carbon of each selected metabolite: coefficient of determination *ρ* for each multiplet, the percentage contribution for each component of the multiplet, the distance of the assigned multiplet from the library shift, a zoomed region of the 2D- ^1^H, ^13^C-HSQC NMR spectrum showing the multiplet of interest and the ^1^H shift of the independent component, and an overlay of the experimental 1D- ^13^C column and simulated data. The coefficient of determination *ρ* and distances from the library chemical shifts are colour coded. The colours indicate whether the software considers the assignment and multiplet estimation to be trustworthy (coefficient of determination *≥* 80%), bordeline (70% > coefficient of determination < 80%) or not trustworthy (coefficient of determination *≤* 70%).

### Evaluation of algorithm performance

To evaluate the algorithm performance, the following metric is used. The **coefficient of determination** *ρ* quantifies the percentage of observed variance that can be explained by the linear regression model. Considering that the model structure is supported by the theoretical assumption that each observed multiplet is a linear combination of single multiplet components, *ρ* is indicative of the goodness of the fit. In this work, *ρ* is used to indicate the analysis reliability. If the experimental 1D spectrum is given as **t**( *M ×* 1) and the estimated spectrum as $\hat{t}$ ( *M ×* 1), the coefficient of determination *ρ* can be calculated as follows:


$$\rho =\frac{\sum_{i=1}^{M}{\left(t_{i}-\bar{t}\right)}^{2}-\sum_{i=1}^{M}{\left(t_{i}-{\hat{t}}_{i}\right)}^{2}}{\sum_{i=1}^{M}{\left(t_{i}-\bar{t}\right)}^{2}}\times 100\;(6)$$

where $\bar{t}$ is the mean of **t**, ${\sum_{i=1}^{M}\left(t_{i}-\bar{t}\right)}^{2}$ is the total sum of squares, and ${\sum_{i=1}^{M}\left(t_{i}-\hat{t}\right)}^{2}$ is the unexplained sum of the squared distance between the observations and predictions. The coefficient of determination *ρ* indicates not only the quality of multiplet estimation, but incorporate also information on the correctness of the multiplet assignment. For this reason *ρ* evaluates the overall performance of the algorithm: it indicates if the multiplet has been correctly localised and consequently the linear regression method is able to closely fit the experimental data. If a signal has a poor signal-to-noise ratio or cannot be found at all, the corresponding *ρ* value will be set to 0. Sample reports are provided in the supporting information, including examples of metabolites with very low concentrations where the algorithm fails to assign and analyse the multiplet correctly.

### Validation of the model and analysis of the results

**Figure 5.**
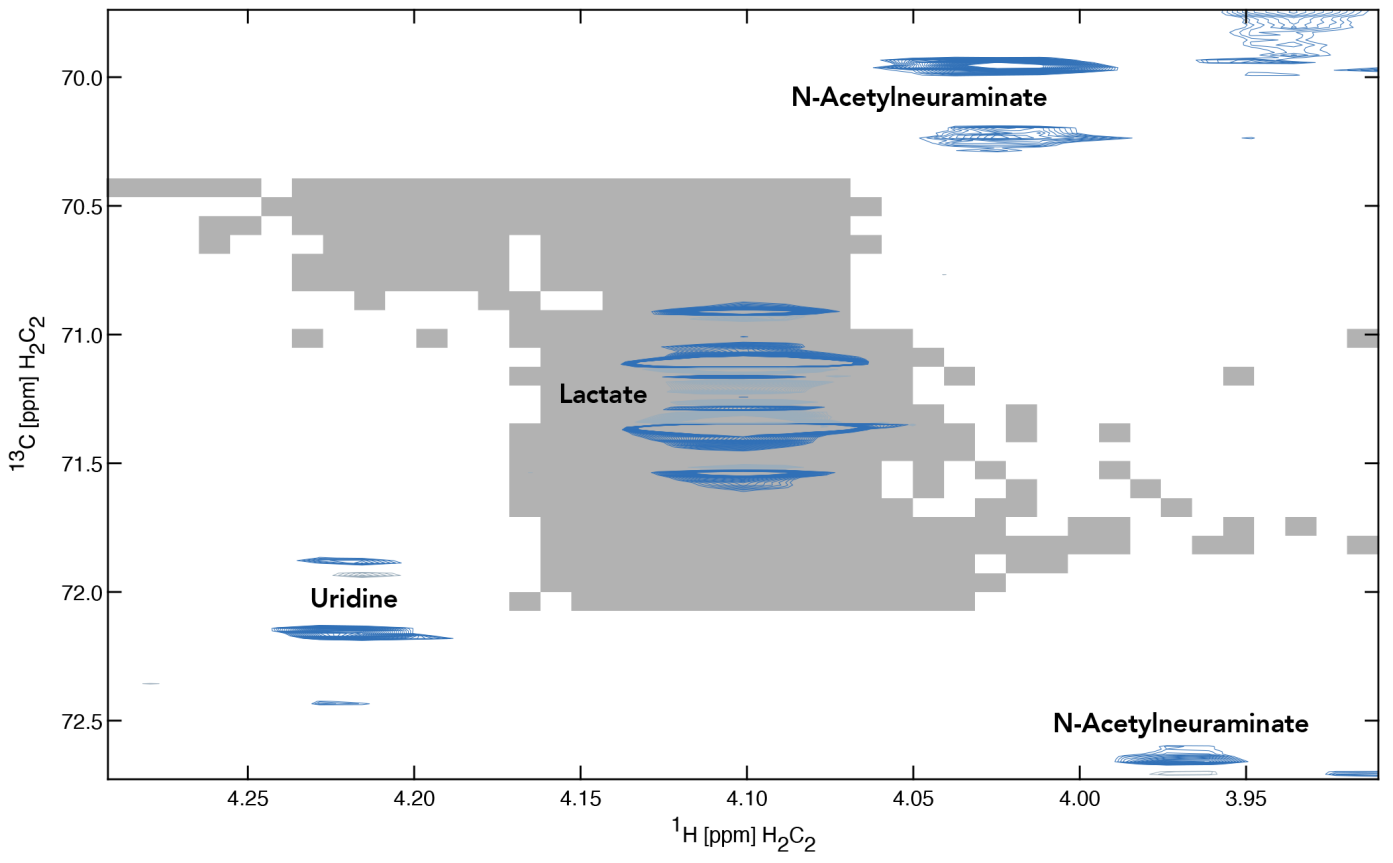
Region of the spectrum showing the multiplet peak for lactate C(2). The gray area underlying the spectrum indicates the highest coefficient of determination computed for lactate C(2) as a function of the chemical shift offset.

***Overall algorithm evaluation.*** The performance of the algorithm developed in this work is evaluated by reporting the results of multiple testing scenarios using different datasets. We manually inspected the datasets to evaluate the software output and in particular to confirm the following outcomes: a) the multiplet is not correctly assigned and the software warns the user about the unreliability of the output (i.e. low coefficient of determination, the distance from the library shifts exceeds the set threshold) b) the multiplet is not correctly identified and the software fails in flagging the issue to the user, giving as output a high coefficient of determination. The coefficient of determination *ρ* is generally affected by the noise and peaks surrounding the multiplet being analyzed, as demonstrated in Figure 5 and Supporting Information Figures S1-S5. Therefore, *ρ* is thought to provide not only a measure of the goodness of the fit but also information about the signal-to-noise ratio (SNR). In heavily congested areas or for signals with poor SNR the computed value of *ρ* is likely to be low. In this case, the user is also warned and the software decision making may be checked manually if desired.

Analysis was performed on four metabolites (alanine, aspartate, glutamate and lactate) on all 13 test 2D- ^1^H, ^13^C-HSQC NMR spectra. In total three different and previously published datasets were used. The three datasets contain a total of 13 2D- ^1^H, ^13^C-HSQC NMR spectra. All NMR data is available via public data repositories. The first dataset contains 2D- ^1^H, ^13^C-HSQC NMR spectra with [1,2- ^13^C] glucose as metabolic tracer (MTBLS241 ^8^). This dataset contains spectra from three biological replicates. The second dataset contains 4 different 2D- ^1^H, ^13^C-HSQC NMR spectra from one sample, but with different amounts of apparent signal-splitting due to J-coupling (eqhn3 ^27^). The third dataset contains 6 2D- ^1^H, ^13^C-HSQC NMR spectra from one sample using different amounts of sampling in a non-uniform sampling scheme (qtmge ^26^). The characteristics of each dataset are summarised in Supporting Information Table S2

The proton and carbon chemical shifts *ρ* values and percentage contribution of each multiplet component for these are given in Supporting Information Tables S3-S5. A quantitative analysis of the results is provided below for each dataset.

**Dataset 1 - CANMS ( MTBLS241)** Manual inspection of the automated analysis reveals that all the 33 metabolites multiplets are correctly identified and localised. The average coefficient of determination *ρ* is 94.06 *±* 5.08%.

The automatic peak-picking is compared against the manual procedure of dataset MTBLS241 to assess the quality of the automated algorithm and compute the accuracy (see figure S6).

**Dataset 2 - Scaling of apparent scalar couplings ( qtmge)** Manual inspection of the automated analysis reveals that all the 44 metabolites multiplets are correctly identified and localised. The average coefficient of determination *ρ* is 95.47 *±* 7.20%. Compared to the previous dataset, the slightly higher standard deviation is due to alanine (C2) ( *ρ* = 56.7%). The algorithm is compatible with the technique of enhancement of splitting due to J-coupling ^27^ meaning that the automated assignment can be used in conjunction with rapidly collected data on high field spectrometers. The degree of splitting enhancement is detected by the software and algorithm parameters automatically altered. The average coefficients of determination for the different values of enhancement of splitting due to J-coupling (1, 2, 4, 8) are *ρ* = 96.14 *±* 3.96%, *ρ* = 97.92 *±* 1.67%, *ρ* = 96.54 *±* 3.65%, *ρ* = 91.30 *±* 12.78% respectively. The results show that a splitting enhancement of 4 is dealt with effectively by the algorithm but an enhancement of 8 is not recommended as it can lead to poorer estimations on less intense multiplets.

**Dataset 3 - NUS-sampling ( eqhn3)** The algorithm gives good results with data collected with reduced sampling down to 25%. At sampling of below 25% the signal to noise is decreased such that only intense peaks can be analysed with confidence and the resulting coefficient of determination of weaker peaks is low. We therefore do not recommend collected data with sampling of less than 25%.

50 out of 66 metabolites multiplets have been picked correctly by the software. The average coefficient of determination (80.47 *±* 20.98%) is significantly lower compared to the previous results. This is due to aspartate, whose co-efficient of determination is low in all the replicates due to poor signal-to-noise ratio because of very load aspartate concentrations. Considering the 16 multiplets that have not been correctly picked, 12 out of 16 have corresponding low coefficient (red flag). In this case the user is warned about the software not being able to provide a reliable analysis and we therefore consider this as a positive outcome for the software (true negative). Instead, 4 multiplets have wrongly been picked by the software and the corresponding coefficients of determination are > 85% (false positive). This last case arises for glutamate. The method described here localises a metabolite along the proton dimension by relying on the cross-correlation metric, as explained in the *Algorithm development* section. With well defined and resolved peaks the peak is easily identified by the algorithm as shown by the large area with a high coefficient of determination for instance with Lactate C(3) (Supporting Information Figure S2). However, if two or more metabolites have multiplets with almost identical patterns and carbon shift, as seen with C(2) (Supporting Information Figure S3) and C(3) (Supporting Information Figure S4) of glutamine and glutamate, the algorithm occasionally fails to correctly pick the metabolite as each metabolite assignment is carried out without knowledge of other metabolites’ assignments. This can almost always be identified by the larger than expected deviation of the chemical shifts from the library values. In one of our test cases the downfield ^1^H of glutamate C(3) is misassigned to glutamine C(3) while the upfield ^1^H is correctly assigned. The misassigned resonance can again be identified by the larger deviation from the library shift and thus analysis can be limited to the correctly assigned ^1^H resonance. The misassignment is due to the glutamine being unlabelled and thus the ease of the algorithm fitting a singlet in close proximity to the library shifts. Care must be taken in the analysis of spectra with significant signals from glutamate and glutamine. Where a carbon has two attached protons, as with aspartate C(3) and glutamate C(3), the algorithm may select the same multiplet independent of the actual ^1^H chemical shift. As the ^13^C/ ^13^C-J coupling constants and the ^13^C chemical shift and the multiplet composition are all identical for the two protons, this can be ignored. If the sample preparation results in the collected data differing significantly from the library shifts the algorithm can adapt in most cases except where the coupling constants of a nearby resonance match that of the actual metabolite. This is shown in Supporting Information Figure S3 where if the sample conditions results in a 0.1 ppm ^1^H shift then a glutamate (C2) can be misassigned as creatine with a high coefficient of determination. However, the rest of the dataset would then show very poor algorithm results allowing the user to realise that there may be an issue with the sample. If proper sample preparation is performed then such problems should not be an issue.

## Conclusion

In this article, we have described a data-driven algorithm which functions within the existing MetaboLab software. The algorithm automatically assigns, annotates and analyses multiplets arising from ^13^C - ^13^C scalar couplings present in 2D- ^1^H, ^13^C-HSQC spectra collected to study metabolism using isotopic tracers. The method is robust and operates with a high degree of accuracy. We have tested the algorithm on three different datasets and show that the algorithm can analyse data collected with a range of tracers with different labelling, at different frequencies, with multiple biological repeats and with different sampling amounts. All spectra were successfully analysed and the algorithm is also able to correctly identify when enhancement of splitting due to J-coupling has been used and to automatically compensate for this effect prior to analysis. The coefficient of determination of the algorithm in the analysis is displayed conveniently in a colour coded form to aid the user in determining the reliability of the result. The algorithm functions successfully in all but the most challenging of situations allowing extensive analysis to be performed even by those with limited knowledge of the field.

## Data Availability

Underlying data can be accessed at the following data repositories: https://www.ebi.ac.uk/metabolights/MTBLS241/files (MTBLS241 dataset), https://doi.org/10.17605/OSF.IO/93BTZ (qtmge dataset) ^32^ and https://doi.org/10.17605/OSF.IO/BD54T (EQHN3 dataset) ^33^. Extended data is available at: https:doi.org/10.5281/zenodo.7120367 ^31^

## Software availability

•    Software available from: https://www.ludwiglab.org/software-development

•    Source code for the presented algorithm is available from: https://github.com/ludwigc/Automated-HSQC-Multiplet-Analysis

•    Archived source code at time of publication: https:doi.org/10.5281/zenodo.7120367 ^31^

Data and software are available under the terms of the Creative Commons Zero "No rights reserved" data waiver (CC0 1.0 Public domain dedication).

## References

[1] Holmes E Wilson ID Nicholson JK : Metabolic phenotyping in health and disease. *Cell.* 2008;134(5):714–717. 10.1016/j.cell.2008.08.026 18775301

[2] Metallo CM Vander Heiden MG : Understanding metabolic regulation and its influence on cell physiology. *Mol Cell.* 2013;49(3):388–398. 10.1016/j.molcel.2013.01.018 23395269 PMC3569837

[3] Ward PS Lu C Cross JR : The potential for isocitrate dehydrogenase mutations to produce 2-hydroxyglutarate depends on allele specificity and subcellular compartmentalization. *J Biol Chem.* 2013;288(6):3804–3815. 10.1074/jbc.M112.435495 23264629 PMC3567635

[4] Lewis CA Parker SJ Fiske BP : Tracing compartmentalized nadph metabolism in the cytosol and mitochondria of mammalian cells. *Mol Cell.* 2014;55(2):253–263. 10.1016/j.molcel.2014.05.008 24882210 PMC4106038

[5] Metallo CM Walther JL Stephanopoulos G : Evaluation of 13c isotopic tracers for metabolic flux analysis in mammalian cells. *J Biotechnol.* 2009;144(3):167–174. 10.1016/j.jbiotec.2009.07.010 19622376 PMC3026314

[6] Walther JL Metallo CM Zhang J : Optimization of 13c isotopic tracers for metabolic flux analysis in mammalian cells. *Metab Eng.* 2012;14(2):162–171. 10.1016/j.ymben.2011.12.004 22198197 PMC3586220

[7] Wiechert W Nöh K : Isotopically nonstationary metabolic flux analysis: complex yet highly informative. *Curr Opin Biotechnol.* 2013;24(6):979–986. 10.1016/j.copbio.2013.03.024 23623747

[8] Chong M Jayaraman A Marin S : Combined analysis of nmr and ms spectra (canms). *Angew Chem Int Ed Engl.* 2017;56(15):4140–4144. 10.1002/anie.201611634 28272839

[9] Xia J Bjorndahl TC Tang P : MetaboMiner--semi-automated identification of metabolites from 2D NMR spectra of complex biofluids. *BMC Bioinformatics.* 2008;9:507. 10.1186/1471-2105-9-507 19040747 PMC2612014

[10] Wang C Timári I Zhang B : COLMAR Lipids Web Server and Ultrahigh-Resolution Methods for Two-Dimensional Nuclear Magnetic Resonance- and Mass Spectrometry-Based Lipidomics. *J Proteome Res.* 2020;19(4):1674–1683. 10.1021/acs.jproteome.9b00845 32073269

[11] Fino R Byrne R Softley CA : Introducing the csp analyzer: A novel machine learning-based application for automated analysis of two-dimensional nmr spectra in nmr fragment-based screening. *Comput Struct Biotechnol J.* 2020;18:603–611. 10.1016/j.csbj.2020.02.015 32257044 PMC7096735

[12] Kuhn S Tumer E Colreavy-Donnelly S : A pilot study for fragment identification using 2d nmr and deep learning. *arXiv preprint arXiv:2103.12169*,2021. 10.48550/arXiv.2103.12169 34480494

[13] Ludwig C Günther UL : MetaboLab--advanced NMR data processing and analysis for metabolomics. *BMC Bioinformatics.* 2011;12(1):366. 10.1186/1471-2105-12-366 21914187 PMC3179975

[14] Alpaydin E : Introduction to machine learning.MIT press,2014. Reference Source

[15] Wishart DS Tzur D Knox C : Hmdb: the human metabolome database. *Nucleic Acids Res.* 2007;35(Database issue):D521–D526. 10.1093/nar/gkl923 17202168 PMC1899095

[16] Wishart DS Knox C Guo AC : Hmdb: a knowledgebase for the human metabolome. *Nucleic Acids Res.* 2009;37(Database issue):D603–D610. 10.1093/nar/gkn810 18953024 PMC2686599

[17] Wishart DS Jewison T Guo AC : Hmdb 3.0—the human metabolome database in 2013. *Nucleic Acids Research.* 2013;41(Database issue):D801–D807. 10.1093/nar/gks1065 23161693 PMC3531200

[18] Wishart DS Feunang YD Marcu A : Hmdb 4.0: the human metabolome database for 2018. *Nucleic Acids Res.* 2018;46(D1):D608–D617. 10.1093/nar/gkx1089 29140435 PMC5753273

[19] Comon P : Independent component analysis, a new concept? *Signal Process.* 1994;36(3):287–314. 10.1016/0165-1684(94)90029-9

[20] Levitt MH : Spin dynamics: basics of nuclear magnetic resonance.John Wiley & Sons,2001. Reference Source

[21] Hyvärinen A : Fast and robust fixed-point algorithms for independent component analysis. *IEEE Trans Neural Netw.* 1999;10(3):626–634. 10.1109/72.761722 18252563

[22] Smith SA Levante TO Meier BH : Computer simulations in magnetic resonance. an object-oriented programming approach. *J Magn Reson A.* 1994;106(1):75–105. 10.1006/jmra.1994.1008

[23] Williamson MP : Using chemical shift perturbation to characterise ligand binding. *Prog Nucl Magn Reson Spectrosc.* 2013;73:1–16. 10.1016/j.pnmrs.2013.02.001 23962882

[24] Johnson AW Jacobson SH : A class of convergent generalized hill climbing algorithms. *Appl Math Comput.* 2002;125(2–3):359–373. 10.1016/S0096-3003(00)00137-5

[25] Sellick CA Hansen R Stephens GM : Metabolite extraction from suspension-cultured mammalian cells for global metabolite profiling. *Nat Protoc.* 2011;6(8):1241–1249. 10.1038/nprot.2011.366 21799492

[26] Jeeves M Roberts J Ludwig C : Optimised collection of non-uniformly sampled 2d-hsqc nmr spectra for use in metabolic flux analysis. *Magn Reson Chem.* 2021;59(3):287–299. 10.1002/mrc.5089 32830359

[27] Smith TB Patel K Munford H : High-Speed Tracer Analysis of Metabolism (HS-TrAM) [version 2; peer review: 4 approved]. *Wellcome Open Res.* 2018;3:5. 10.12688/wellcomeopenres.13387.2 29503875 PMC5811808

[28] Orekhov VY Jaravine VA : Analysis of non-uniformly sampled spectra with multi-dimensional decomposition. *Prog Nucl Magn Reson Spectrosc.* 2011;59(3):271–292. 10.1016/j.pnmrs.2011.02.002 21920222

[29] Kazimierczuk K Orekhov VY : Accelerated nmr spectroscopy by using compressed sensing. *Angew Chem Int Ed Engl.* 2011;50(24):5556–5559. 10.1002/anie.201100370 21538743

[30] Delaglio F Grzesiek S Vuister GW : Nmrpipe: a multidimensional spectral processing system based on unix pipes. *J Biomol NMR.* 1995;6(3):277–293. 10.1007/BF00197809 8520220

[31] Ludwig C : ludwigc/AutomatedHSQC-Multiplet-Analysis: Extended Data and Source Code for Automated HSQC Multiplet Analysis (v1.0). *Zenodo.* 2022. 10.5281/zenodo.7120367

[32] Ludwig C Jeeves M Roberts J : Rapid data collection of spectra for the use in tracer based metabolism studies.[Dataset].2020. 10.17605/OSF.IO/93BTZ

[33] Ludwig C : HS-TrAM.[Dataset].2018. 10.17605/OSF.IO/BD54T

