# Supplementary material for: Automated analysis for multiplet identification from ultra-high resolution 2D- 1H, 13C-HSQC NMR spectra
Source: Wellcome Open Res. 2023 May 19;7:262. Originally published 2022 Oct 14. [Version 2] doi: 10.12688/wellcomeopenres.18248.2 (PMC10050905; doi:10.12688/wellcomeopenres.18248.2)
Supplement: Peer review history

# Peer review: Reviewer response for version 1

**DOI:** 10.21956/wellcomeopenres.20227.r54885

**Published:** 2023-03-28

**Authors:** Fuhua Hao

**Affiliations:** 1 Department of Veterinary and Biomedical Sciences and the Center for Molecular Toxicology and Carcinogenesis, The Pennsylvania State University, University Park, PA, USA

In this current paper, the authors developed a data-driven algorithm to automatically annotate and analyze signal multiplets in 2D- ^1^H, ^13^C-HSQC spectra. This work not only described a detailed algorithm development but also tested the algorithm performance with three different datasets. The work should provide a good tool for different metabolic pathway analyses, especially, stable isotope tracing analysis. I should note that I am not an expert on algorithm development, but I think the work is of good quality and recommend indexing. However, I still have a couple of issues for consideration. 

1. Figure 1. Was it a spectrum with a mix of three different samples or an overlap of three different spectra?
2. As mentioned in NMR methodology, the 2D- ^1^H, ^13^C-HSQC experiment time is approximately 4 hours for a sample. This seems impossible to use it for a large number of samples, like 50, 100, or even 200 samples.
3. For the relative intensity of each multiplet component, the authors mentioned “ *vector B is normalized*”, could you give what normalization method was used?
4. The authors also mentioned: “ *Analysis was performed on four metabolites (alanine, aspartate, glutamate and lactate) on all 13 … spectra. Dataset 1 … 33 metabolites multiplets are correctly identified and localised. Dataset 2 … 44 metabolites multiplets are correctly identified and localised. Dataset 3 … 50 out of 66 metabolites multiplets have been picked correctly by the software.*” Here, 33, 44, and 50 out of 66 metabolites multiplets, were they from the four above metabolites?
5. In addition, the authors described: *“if the sample preparation results in the collected data differing significantly from the library shifts the algorithm can adapt in most cases except where the coupling constants of a nearby resonance match that of the actual metabolite*''. So, what proper sample preparation should be suggested before collecting data?
6. " *...poor signal-to-noise ratio because of very load aspartate concentrations*". Here, please correct “load” to “low”.

Is the work clearly and accurately presented and does it cite the current literature?

Yes

If applicable, is the statistical analysis and its interpretation appropriate?

Yes

Are all the source data underlying the results available to ensure full reproducibility?

Partly

Is the study design appropriate and is the work technically sound?

Yes

Are the conclusions drawn adequately supported by the results?

Yes

Are sufficient details of methods and analysis provided to allow replication by others?

Partly

Reviewer Expertise:

NMR, LC-MS, metabolomics, diseases

I confirm that I have read this submission and believe that I have an appropriate level of expertise to confirm that it is of an acceptable scientific standard.

# Response

**Published:** 2023-04-26

**Authors:** Christian Ludwig

**Affiliations:** University of Birmingham, UK

We thank Fuhua Hao for their kind comments and have addressed the issues raised as follows: We amended the legend for Figure 1 to make clear that the plot is an overlay of three different spectra. We also added a comment on how to reduce the data acquisition time by increasing the enhancement of splitting due to J-coupling and linked to a reference. We expanded our explanation of how the vector normalisation was achieved by adding the equation used. We have made clear the source of the multiplets analysed in each dataset to show that they come from the four metabolites discussed (alanine, lactate, glutamate and aspartate). We added a comment on how best to minimise pH variability between samples. We corrected the typing error spotted.

# Peer review: Reviewer response for version 1

**DOI:** 10.21956/wellcomeopenres.20227.r55154

**Published:** 2023-03-24

**Authors:** Iain James Day

**Affiliations:** 1 School of Life Sciences, University of Sussex, Brighton, UK

The manuscript by Ferrante et al. describes an automated analysis procedure for the identification of ^13^C- ^13^C multiplets from high resolution proton-carbon correlation experiments performed on metabolomics samples.

 The use of labelled tracers allows metabolic pathway information to be determined, however, identification of these metabolic products can be challenging. HSQC spectra provide information on CH pairs within a molecule. By incorporating 13C-labelled tracers, additional splittings are observed along the F1 axis arising from 13C-13C couplings in the metabolites. In this sense these experiments contain substantially larger amounts of information that just HSQC spectra of the metabolites. 

 The authors present a framework built upon the Metabolab platform for the identification and quantification of the metabolites. The paper clearly presents the logic of the algorithm developed and provides a good overview of its function and the quality of the results obtained. 

 There is clearly a requirement for high digital resolution in the 13C dimension for this approach to be successful, and the data presented has this. It would be useful for the authors to comment on what the limits of this digital resolution are. What is the minimum digital resolution required in order for the their algorithm to be effective? 

 Additionally they comment on the use of non-uniform sampling schemes. Typically sparse sampling schemes work best for sparse datasets, e.g. better performance with lower sampling schemes for HSQC than NOESY. It would be useful to give a comparison between NUS and fully sampled data on the same sample. 

 There are a couple of minor typos on page 7, column 2, second item, and page 10, column 2, 2nd paragraph

Is the work clearly and accurately presented and does it cite the current literature?

Yes

If applicable, is the statistical analysis and its interpretation appropriate?

Not applicable

Are all the source data underlying the results available to ensure full reproducibility?

Yes

Is the study design appropriate and is the work technically sound?

Yes

Are the conclusions drawn adequately supported by the results?

Yes

Are sufficient details of methods and analysis provided to allow replication by others?

Yes

Reviewer Expertise:

Structure elucidation, data analysis, algorithm development, software development

I confirm that I have read this submission and believe that I have an appropriate level of expertise to confirm that it is of an acceptable scientific standard.

# Response

**Published:** 2023-04-26

**Authors:** Christian Ludwig

**Affiliations:** University of Birmingham, UK

We thank Ian Day for his kind review and comments. We have addressed the issues mentioned as follows: We added a statement suggesting the optimal resolution required for accurate determination of multiplet components and how this can be altered when increasing the amount of enhancement of splitting due to J-coupling. We have added analysis of the fully sampled spectrum within the qtmge dataset. The results are being added to tables S2 and S5. We have corrected the typing errors mentioned.
